# An Update on Detection Technologies for SARS-CoV-2 Variants of Concern

**DOI:** 10.3390/v14112324

**Published:** 2022-10-22

**Authors:** Wenjie Jiang, Wangquan Ji, Yu Zhang, Yaqi Xie, Shuaiyin Chen, Yuefei Jin, Guangcai Duan

**Affiliations:** 1 Department of Epidemiology, College of Public Health, Zhengzhou University, Zhengzhou 450001, China; 2 Henan Key Laboratory of Molecular Medicine, Zhengzhou University, Zhengzhou 450001, China

**Keywords:** SARS-CoV-2, VOCs, PCR, CRISPR

## Abstract

Severe Acute Respiratory Syndrome Coronavirus 2 (SARS-CoV-2) is responsible for the global epidemic of Coronavirus Disease 2019 (COVID-19), with a significant impact on the global economy and human safety. Reverse transcription-quantitative polymerase chain reaction (RT-PCR) is the gold standard for detecting SARS-CoV-2, but because the virus’s genome is prone to mutations, the effectiveness of vaccines and the sensitivity of detection methods are declining. Variants of concern (VOCs) include Alpha, Beta, Gamma, Delta, and Omicron, which are able to evade recognition by host immune mechanisms leading to increased transmissibility, morbidity, and mortality of COVID-19. A range of research has been reported on detection techniques for VOCs, which is beneficial to prevent the rapid spread of the epidemic, improve the effectiveness of public health and social measures, and reduce the harm to human health and safety. However, a meaningful translation of this that reduces the burden of disease, and delivers a clear and cohesive message to guide daily clinical practice, remains preliminary. Herein, we summarize the capabilities of various nucleic acid and protein-based detection methods developed for VOCs in identifying and differentiating current VOCs and compare the advantages and disadvantages of each method, providing a basis for the rapid detection of VOCs strains and their future variants and the adoption of corresponding preventive and control measures.

## 1. Introduction

Severe Acute Respiratory Syndrome Coronavirus 2 (SARS-CoV-2) is a single-stranded positive-sense RNA virus belonging to the β-coronavirus cluster, which has four structural proteins: the spike (S) protein, envelope (E) protein, membrane (M) protein, and nucleocapsid (N) protein [1]. Since the disease named Coronavirus Disease 2019 (COVID-19) first began circulating in December 2019, the SARS-CoV-2 virus has continuously evolved, with numerous variants emerging across the world. These variants are generally categorized as the variant of interest (VOI), variant under monitoring (VUM), and variant of concern (VOC). As of writing, five VOCs have emerged, Alpha, Beta, Gamma, Delta, and Omicron [2]. Although with the development and vaccination of vaccines, the number of confirmed cases and deaths caused by SARS-CoV-2 has not decreased globally, especially with the emergence of the SARS-CoV-2 variants.

Most of the current vaccines are based on the SARS-CoV-2 spike protein, while VOCs contain spike protein mutations; thus, the high transmissibility and mutagenicity may contribute to the continuation of this outbreak. In addition, its role in the mechanism of host immune escape also leads to decreased vaccine effectiveness and neutralization of antibodies against the SARS-CoV-2 strain, posing a threat to global public health security and health [3,4]. Early screening and classification of VOCs can provide a basis for the development of targeted public health precautions to slow the spread of VOCs, thereby further preventing a new round of COVID-19 outbreaks. Reverse transcription-quantitative polymerase chain reaction (RT-PCR) is considered the gold standard assay for diagnosing SARS-CoV-2 infection, but targeting specific detection regions may lead to reduced sensitivity of SARS-CoV-2 variants [5,6].

**Figure 1:**
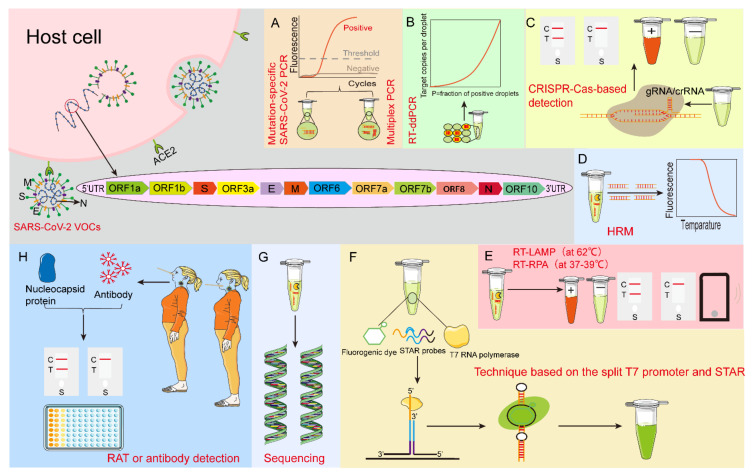
Schematic of nucleotide and protein detection methods for SARS-CoV-2 VOCs. (**A**) Mutation-specific SARS-CoV-2 PCR and multiplex PCR. (**B**) RT-ddPCR: droplet digital reverse transcription-quantitative polymerase chain reaction. (**C**) CRISPR-Cas-based detection technology: Clustered Regularly Interspaced Short Palindromic Repeats-CRISPR-associated proteins-based detection technology. (**D**) HRM: high−resolution melting. (**E**) RT-LAMP: reverse transcription loop-mediated isothermal amplification assay (at 62 °C) and RT-RPA: reverse transcription-recombinase polymerase amplification (at 37–39 °C). (**F**) Technique based on the split T7 promoter and luminescent RNA aptamer (STAR). (**G**) Sequencing. (**H**) RAT: rapid antigen test, and antibody detection. ACE2: angiotensin-converting enzyme 2.

Therefore, based on the results of recently published studies, this review summarized various detection technologies developed for the current SARS-CoV-2 variants, especially VOCs, such as: mutation-specific SARS-CoV-2 PCR, multiplex PCR, loop-mediated isothermal amplification assay (LAMP), Clustered Regularly Interspaced Short Palindromic Repeats-CRISPR-associated proteins (CRISPR-Cas)-based detection technology, droplet digital RT-PCR (RT-ddPCR), high−resolution melting (HRM), reverse transcription-recombinase polymerase amplification (RT-RPA), a technique based on the split T7 promoter and luminescent RNA aptamer (STAR), viral genome sequencing, rapid antigen test (RAT) and antibody detection, etc. (Figure 1). This review analyzed the advantages and disadvantages of each detection technique to improve the accuracy of SARS-CoV-2 variants detection and prevent false negative results. Secondly, it provided a theoretical basis for improving the effectiveness of epidemiological surveillance strategies, researching and developing new detection technologies for variants, and targeting and rapid selection of new SARS-CoV-2 variants detection technology to prevent outbreaks and epidemics as much as possible in the future.

## 2. Nucleic Acid Detection-Based Test

RT-PCR is the widely used method for rapid detection of SARS-CoV-2, but the advent of variants can evade the diagnosis of existing RT-PCR techniques [6,7]. Whole genome sequencing (WGS) is the reference standard for detecting SARS-CoV-2 variants, but it is costly and has a long turnaround time. Furthermore, WGS does not provide good data for genome sequencing of samples with low viral loads [8,9]. These limitations have prompted research into improving methods of PCR for identifying SARS-CoV-2 variants. These include, for example, mutation-specific SARS-CoV-2 PCR, multiplex PCR, LAMP assay, CRISPR-Cas-based detection technology, etc.

### 2.1. Mutation-Specific SARS-CoV-2 PCR

Mature RT-PCR is widely used in the detection of SARS-CoV-2, and in order to exploit its advantages to identify variants quickly and accurately, a mutation-specific RT-PCR detection technique based on currently known VirSNiP mutations was designed. Two amino acids deletions (ΔE156/ΔF157) of S-gene target failure (SGTF) reported by RT-PCR were utilized as a rapid screening method for Delta strains. Moreover, this research method has high sensitivity and specificity, and binding to N1 (nucleocapsid gene), N2, and RP (human RNase P) genes can achieve pre-screening of the Delta variant [10]. In addition, primers and probes for E484K and N501Y in the S gene were developed in previous research and combined with open reading frame (ORF) 1ab to form duplex rRT-PCR detection Alpha and Beta, respectively, which had good detection capabilities [11]. The Omicron variant, as the major prevalent strain, also has infectivity and immune escape capacity. It has been found that identification of Omicron could be achieved using partial ORF1ab gene target failure (pOGTF). pOGTF was defined as the difference in CT value between the E gene target and the ORF1ab gene target (dEO), with a sensitivity of 98.6% and specificity of 99.9% based on dEO outliers for BA.2.12.1 compared to the WGS [12]. Since most techniques cannot effectively distinguish between Delta and Omicron variants, according to the gene sequence analysis results of Omicron and Delta variants, primers and probes were designed for Omicron and wild type based on Δ31−33 aa deletion at 9-bp in the N gene, and for Delta and wild type based on Δ157−158 aa deletion at 6-nt in the S gene. The detection limits of Omicron, Delta, and wild type were 14.3, 32.0, and 21.5 copies per PCR reaction, respectively [13].

Given that the SARS-CoV-2 variants have different mutation characteristics, RT-PCR designed for primers with known mutant properties can detect variants more quickly. Table 1 shows some research about RT-PCR techniques that were developed for mutation sites to identify the corresponding VOCs.

Mutation-specific RT-PCR only can recognize currently known VOCs. Since this method designs primers according to the characteristic mutations of VOCs, it has higher sensitivity and lower average cost and can be used as a pre-screening for VOCs. However, because the assay requires a deeper understanding of the nature of specific mutations in various variants based on gene sequencing results, the applicability of the method for possible future variants needs to be improved. So WGS is still an essential tool for emerging variants.

### 2.2. Multiplex PCR

The principle of multiplex PCR is similar to that of RT-PCR, except that multiple pairs of specific primers and probes are added to the reaction tube to detect multiple pathogens at the same time quickly. Therefore, compared with mutation-specific RT-PCR, multiplex PCR can unknowingly simultaneously detect multiple mutation sites of SARS-CoV-2 in a single reaction and rapidly identify current VOCs.

Multiplex quantitative RT-PCR technique for single nucleotide was reportedly designed based on molecular beacons for different mutations in the SARS-CoV-2 spike protein (d69–70, K417N/T, L452R, T478K, E484K/Q, N501Y, and E484A) to identify current VOCs (Alpha, Beta, Gamma, Delta, and Omicron). Moreover, due to the superior selectivity, specificity, and self-extinguishing properties of the molecular beacons, other mutations in the target region do not trigger a false-positive signal but may lead to a false-negative signal. However, it is undeniable that molecular beacons can be an ideal tool for differentiating point mutations on account of their characteristics that can be designed and produced within two to three weeks [28]. In addition, a quadruple rRT-PCR method containing four primer pairs and locked nucleic acid (LNA) nucleotide fluorescent probes was developed in the study of Durand et al. and allowed faster detection of relevant variants in a single reaction, such as Alpha strains. This method reduces the cost of analyzing all samples per day and is three times cheaper than individual RT-PCR but requires prior knowledge of the mutation site properties of the SARS-CoV-2 variants [29]. Studies have found that multiplex tandem PCRs can also detect current VOCs simultaneously. Moreover, the turnaround time (TAT) of this method is shorter than that of the WGS, taking approximately three hours [30,31].

To rapidly detect the SARS-CoV-2 variants for epidemiological monitoring, Mertens et al. proposed a rapid detection algorithm. It used SGTF-sensitive multiplex RT-qPCR by targeting six specific mutations (N501Y, ΔHV 69/70, E484K, H655Y, L452R, and P681R) probe PCR melting curve to analyze melting temperature (Tm) for SARS-CoV-2 variants typing and monitoring [32]. Furthermore, since the combination of multiplex PCR and melt curve analysis can produce results more quickly, new mutation detection methods, such as multicolor melting curve analysis (MMCA) and MeltArray assay, can be added to track and monitor variation, depending on the number of variant mutations and realistic needs, so this advantage is fully used by researches to identify Alpha, Beta, Gamma, Delta, and Omicron subvariants [33,34,35]. Although genotyping methods based on single nucleotide polymorphisms (SNPs) have been widely developed and used, these methods need to be adjusted before the application due to the emergence of new variants, and this time delay is important to control virus transmission. To address this problem, researchers developed a multiplex fragmentation analysis method (CoVarScan). This method involved RT-PCR amplification with fluorescently labeled primers, followed by amplification products with different sizes and fluorescent colors that were resolved and analyzed with capillary electrophoresis and identified fragments based on their different combinations. In this study, fluorescently labeled primers were designed for five frequently deleted regions (RDR; S: RDR1, S: RDR2, S: RDR3–4, ORF1A, and ORF8) and three SNPs (S: N501Y, S: L452R, and S: E484K), and multiplex combinatorial analysis of the fragments were able to distinguish all VOCs, which showed 96% sensitivity and 99% specificity compared to WGS. So far, CoVarScan has distinguished all VOCs without modification, and therefore, this novel method based on fragment analysis is well suited to detect new variants, although the sensitivity is low compared to some RT-PCR methods [36].

At present, the SARS-CoV-2 virus is constantly mutating, and rapid identification of mutation types can warn of early infection and then implement public health interventions to quickly control infections and take targeted treatment measures according to the corresponding variant types, which can effectively prevent the spread of COVID-19 as much as possible. Because multiplex PCR can detect multiple mutations simultaneously, it can be used for the diagnosis of SARS-CoV-2 mutations and VOCs and achieve the purpose of taking prevention and control measures to control the spread of the epidemic quickly. Moreover, in practice, mutation-specific RT-PCR and multiplex PCR are often used synergistically because of the many similarities between them.

### 2.3. LAMP Assay

LAMP assay exponentially amplifies nucleic acids at a constant temperature, with no instrumentation required after the reaction, and the visual color change method can be used to know the detection results. Due to its advantages in instrument requirements, detection efficiency, operating methods, and result interpretation, LAMP is well suited for use in resource-limited environments and is an alternative technique to RT-PCR [37].

It was reported that the PS6 primer based on the SARS-CoV-2 N gene was designed and optimized for LAMP, and Calcein, a metal ion indicator, was added to the reaction system to determine the detection results according to the color change in the reaction tube. Since this primer was located in a highly conserved region of the N gene from 12 to 213-nt, the study demonstrated good compatibility with Alpha, Beta, Gamma, Delta, and Omicron and as a reliable visual assay for the diagnosis of SARS-CoV-2 variants [38]. In addition, colorimetric RT-LAMP based on N gene or N2/E1 primer set as targets has been developed to successfully identify Gamma, Zeta, Delta, B.1.1.374, and B.1.1.371, with high sensitivity and specificity, and this technology did not require RNA extraction, and no need additional steps and equipment, which is a highlight of current sample collection and detection [39]. The fluorescent readout-based PCR technique has a drawback, and the non-specific binding of SYBR fluorescent dye to dsDNA leads to false-positive results. In order to eliminate this non-specific amplification and improve the sensitivity and specificity of the assay, fluorescence-quenched reverse transcription loop-mediated isothermal amplification (FQ-LAMP) was developed. The method was amplified using fluorescently labeled reporter gene primer, LoopB primer (FLB), and short complementary oligonucleotide quenching agent (QLB) in a real-time thermal cycler for 30 min at 65 °C. Next, the reaction was cooled to room temperature to read the fluorescence signal, and three VOCs, Alpha, Delta, and Omicron, were identified. Because the assay results can be interpreted with the naked eye or a mobile phone, FQ-LAMP is more suitable for point-of-care (POC) or home testing environments than the standard RT-LAMP [40]. Given that PCR-based diagnostics require the use of complex laboratory instruments and time-consuming steps, these requirements limit the transition of technology to medical points and fail to address accessibility in under-resourced areas, but the proposed microfluidic analysis device enables the reliability of developed immediate care devices. The assay was based on isothermal RT-LAMP amplification, utilized the advantages of SGTF, and combined binary detection systems based on spatial separation of N gene and S gene-specific primers, additive manufacturing (AM) technology with smartphone-based optical readout to achieve the detection of Alpha variant. Wherein the sensitivity was greater than 90%, the specificity was equal to 100%. Because the additive manufacturing process can quickly create molding and unnecessary expensive tools and can be completed in a matter of weeks, the POC unit is suitable for mass production, reducing the overall cost of sample collection, testing, and analysis, promising universal access to resource-poor settings [41]. Furthermore, in order to distinguish between wild-type and mutant types and improve mutant differentiation specificity, Talap et al. added NED and FAM-labeled one-step displacement (OSD) probes to RT-LAMP to achieve the detection of single-site differences in the SARS-CoV-2 S gene, such as disguising samples with or without P681R mutation [42]; and Yang et al. designed a single loop primer (LF or LB) close to the R203M-containing region instead of a pair of loop primers (LF and LB) in the RT-LAMP assay based on the R203M mutation to achieve detection of SARS-CoV-2 and Delta recognition within 1 h [43].

In summary, LAMP may be used for the detection of SARS-CoV-2 and its variants in the COVID-19 pandemic due to the fact that LAMP can use internal primers, external primers, and loop primers simultaneously, making it highly efficient amplification and highly sensitive, as well as having advantages of simplicity, affordability, speed, and readability of experimental results.

### 2.4. CRISPR-Cas-Based Detection Technology

Clustered Regularly Interspaced Short Palindromic Repeats (CRISPR) and CRISPR-associated proteins (Cas), CRISPR-Cas, the diagnostic principle is that CRISPR RNA (crRNA) or guide RNA (gRNA) can specifically bind to the target nucleic acid sequences; when the Cas protein forms an effector complex with crRNA or gRNA and the target sequence, the cis-cleavage activity and trans-cleavage activity of Cas protein are activated, the reported probe is cut, and the report signal is released to achieve the detection effect. CRISPR-Cas nucleic acid diagnostic technology has been widely studied and applied in recent years because of its advantages of good specificity, high sensitivity, simple operation, and short detection time. As a result, CRISPR-Cas technology promises to be a fast, accurate, and portable POC diagnostic [44].

The lateral flow assay (LFA) has been successful in diagnosing many diseases over the past few decades and has become popular among healthcare providers, especially those with limited resources, as well as families requiring personal health monitoring [45]. Therefore, in several studies, due to the high sensitivity of Cas9 to small changes in the target sequence, a Cas9-based CRISPR LFA was developed to design sgRNAs corresponding to mutation sites that could represent SARS-CoV-2 VOCs, which can effectively identify VOCs (Alpha, Beta, and Gamma) without sequencing for diagnosis [46,47]. Further, given the specificity of crRNA and gRNA to the target sequence, CRISPR-Cas12a diagnostic techniques designed for the synthesis of mismatch crRNAs or gRNA mutation sites for SARS-CoV-2 variants have been applied to the detection and identification of SARS-CoV-2 variants, such as Alpha, Beta, Gamma, Delta, Kappa, Lambda, and Epsilon variants [48,49,50,51,52,53,54]. Furthermore, these assays used a two-pot strategy, a pre-amplification step prior to CRISPR-Cas detection, which increased reaction time and carryover contamination. Therefore, in an earlier study, taking advantage of the high specificity of BrCas12b and its powerful trans-cleavage activity at RT-LAMP reaction temperature, the combination of CRISPR-BrCas12b and RT-LAMP was applied to a one-pot detection reaction system, coined CRISPR-SPADE. It could identify SARS-CoV-2 VOCs in 10–30 min, including Alpha, Beta, Gamma, Delta, and Omicron, with sensitivity, specificity, and accuracy of 92 ± 8%, 99 ± 4%, 96 ± 7%, respectively. Additionally, a pot of reagents was freeze-dried to facilitate transportation, distribution, and deployment to meet the needs of cryogenic logistics in remote or harsh environments [55]. In addition, given that there is no POC detection method to effectively and specifically identify SARS-CoV-2 variants, Arizti-Sanz et al. designed crRNA and RPA primers for spike protein-specific mutations and developed a Cas13-based nucleic acid diagnostic procedure for SINEv2 distinguishing between Alpha, Beta, Gamma, and Delta in 90 min. Importantly, FastAmp lysis reagent and 5% RNase inhibitors were operated at room temperature for 5 min to process rapidly and inactivate the virus sample; secondly, the use of equipment-free, freeze-dried, and ambient-temperature sample lysis method added user-friendliness [56]. Using similar principles, Lin et al. combined isothermal recombinase-aided amplification for single-nucleotide mutations and CRISPR-Cas12a to achieve Alpha, Beta, and Delta variants and Omicron subline BA.1 and BA.2 detection. The sensitivity was 100.0%, and the specificity was 94.9–100.0% [57]. A highly sensitive portable field assay for HV69–70del mutations based on CRISPR/Cas13a system was also investigated, which could detect 1 copy/μL of Alpha and Omicron variants [58].

This CRISPR-Cas-based genotyping technology is suitable for POC diagnosis because equipment, reagents, and facilities can be shared, can greatly save costs, reduce detection time, and visualize and read test results. Hence, CRISPR-Cas-based technology is becoming a diagnostic tool for rapid and immediate detection of SARS-CoV-2 variants and epidemiological surveillance.

### 2.5. Other Nucleic Acid Detection-Based Test

RT-ddPCR divides the reaction system containing nucleic acid monodisperse into highly monodisperse droplets, performs PCR reactions on each droplet, and then obtains the starting copy number or concentration of the target molecule according to the Poisson distribution principle and the number and ratio of positive droplets instead of Ct values [59]. Compared to RT-PCR, RT-ddPCR does not rely on a standard curve and Ct, making results more accurate, fast, and absolutely quantitative so that SARS-CoV-2 mutations can be detected with high sensitivity, specificity, and quantity. In some research on the characteristic mutations of SARS-CoV-2 VOCs in wastewater, RT-ddPCR was used to specifically detect mutational signatures, such as Alpha, Beta, Gamma, Delta, Mu, Lambda, and Omicron, and could monitor the prevalence trend of mutations in wastewater samples, more importantly, future SARS-CoV-2 variants [60,61]. In 419 positive clinical samples, 99.7% of the sample mutations detected by RT-ddPCR were consistent with the results of WGS, and the Omicron variant was precisely detected [62]. A study found that the characteristic mutations of SARS-CoV-2 VOCs in 547 wastewater samples were detected by RT-ddPCR and amplicon-based sequencing and found that 42.6% of the positive samples detected by RT-ddPCR were missed by sequencing, and 26.7% of the negative samples tested by sequencing were positive by RT-ddPCR. It can be seen that the sensitivity of RT-ddPCR to detect mutations is higher than that of sequencing [63]. Therefore, care should be taken when using sequencing to detect mutations in wastewater environments.

HRM is a molecular biology method for analyzing PCR amplification products based on differences in the Tm between purine and pyrimidine bases. In order to verify its effectiveness in the detection of the Omicron variant, it has been reported that the cDNA of wild SARS-CoV-2 receptor-binding domain (RBD) and SARS-CoV-2 RBD variants were obtained by RT-PCR amplification, and then the primers of Omicron BA.1 specific G339D and D796Y mutations were designed, and nested PCR amplification was performed according to the first RT-PCR amplification products; finally, the amplicons were analyzed by HRM. The results showed that the HRM curve and Tm values were consistent with those of the cDNA amplicons of the Omicron variant RBD. In addition, the method can be adapted for the detection of future SARS-CoV-2 variants by designing sufficient oligonucleotides [64].

LAMP requires the use of a heating device to keep the temperature at 62 °C, but RPA is amplified at 37–39 °C, which can be achieved by using methods such as water baths, hand warmers, or body temperatures. It is a simple and inexpensive method, which is ideal for use in resource-constrained environments [65]. The E and RdRP genes of SARS-CoV-2 were determined with real-time fluorescent RT-RPA, similar to the method of using Cq value as viral load; the threshold time (TT) of the RT-RPA results was converted to viral load equivalents as a semi-quantitative method. The results showed that the sensitivity and specificity of SARS-CoV-2 positive strains were 96% and 97%, respectively, compared with RT-PCR, and four VOCs (Alpha, Beta, Delta, and Omicron) were also detected. Although no data on the robustness of the anti-variant SARS-CoV-2 detection method for VOCs detection was provided in this study, the role of this method in the detection of VOCs was confirmed. Importantly, since new variants will continue to emerge unpredictably, this is an alternative to reduce the false negatives of RT-PCR assays [66].

The REVELER two-color competitive binding assay technology: a multi-component DNA enzyme generates a signal for a specific mutant nucleic acid sequence labeled with a fluorescent dye, signal amplification, and cleavage of the quenched fluorescent reporter gene. It could achieve 100% detection of variants. Compared with CRISPR-Cas, it had good advantages in POC diagnosis due to its extensive targeting, reducing the fluorescence background value, and avoiding viral or bacterial contamination [67].

In addition, to address the challenges of optimizing multiple enzyme reactions by most isothermal amplification methods and the inadequacy of the initial denaturation/annealing steps, an isothermal transcriptional amplification technique based on the split T7 promoter and STAR signal amplification technique was developed. The ternary linkage structure used in this method could easily distinguish changes in the target sequence, and the target RNA could be detected using a single enzyme, enabling the detection of the SARS-CoV-2 N gene in no more than 30 min at 37 °C. Furthermore, multiplex STAR was established to develop mango aptamer and malachite green aptamer for the D614G mutation in the N and S genes, respectively, and to distinguish wild-type and D614G mutants (Alpha, Beta, Delta, and Omicron) based on the change in aptamer color. This approach eliminates the need for expensive fluorophore labeling and nucleic acid purification [68].

## 3. Viral Genome Sequencing

WGS is the best way to identify SNPs of the SARS-CoV-2 variants, but the high cost prevents the practicality and generalizability of this method. As a result, some methods in generation sequencing, next-generation sequencing (NGS), and third-generation sequencing are beginning to be used gradually to detect SARS-CoV-2 variants.

### 3.1. Generation Sequencing: Sanger Sequencing

Sanger sequencing, as a traditional method, utilizes DNA polymerase selective incorporation of fluorescently labeled dideoxynucleotides, enabling the prolonged oligonucleotides to be selectively terminated at G, A, T, or C bases. It has the advantages of being easy to use, fast, and low cost; facilities for Sanger sequencing infrastructure are available in many laboratories. Primers were designed to target the spike protein gene region as described previously, sequence information of this region was obtained by Sanger sequencing, and current VOCs could be identified based on changes in the gene sequence information [69,70,71]. It is considered that the SARS-CoV-2 genomic RNA is too large for Sanger sequencing to analyze the entire genome. Therefore, one study performed long-range RT-PCR amplification of the entire SARS-CoV-2 S gene, including a 4 kb region, and then the entire S gene sequence was analyzed by Sanger sequencing. In the study, the authors compared the analysis results with the reference SARS-CoV-2 genome, identified amino acid mutations in the sequence, and determined the type of VOCs (Alpha, Delta, and Omicron) according to the nature of mutations, which could be used to track and monitor SARS-CoV-2 variants with conventional Sanger sequencing instruments [72].

### 3.2. NGS: Illumina Sequencing

Testing the SARS-CoV-2 variants in wastewater samples also is a valuable and effective way to indicate the prevalence of COVID-19. However, most of the variants in wastewater are low-abundance mutations, for which studies provided a highly sensitive method for the detection of low-frequency variants, namely Illumina sequencing. It can identify VOCs by detecting low-frequency variants (LFVs) and branch-defined mutations of specific minimum allele frequency (AF) targeted sequencing by SARS-CoV-2 variants [73]. Moreover, ADSSpike, improved and developed for Illumina Sequencing, is a practical and cost-effective detection tool. It can reduce the number of PCR cycles and identify characteristic SNPs from S genes associated with SARS-CoV-2 variants based on amplicon depth sequencing [74].

### 3.3. Third Generation Sequencing: Nanopore Sequencing

Nanopore sequencing combined with mutation-specific PCR enabled the identification of samples with very low or negative viral loads and allowed rapid, highly sensitive determination of VOCs. More importantly, the method can identify new SARS-CoV-2 variants that may emerge in the coming years due to gene sequencing [75]. Compared with short-read sequencing technologies such as Illumina sequencing, long-read nanopore sequencing is more effective for smaller batches of samples and has shorter turnaround times. However, the accuracy of nanopore sequencing for the SARS-CoV-2 variants is limited [76].

WGS and NGS sequencing are emerging sequencing techniques. Although NGS is widely used, there is a high proportion of uncertainty in the computational errors and biases in NGS [77]. Secondly, dedicated and expensive sequencing equipment is required, and the readout process of these devices is relatively slow and costly. But NGS-based testing can meet the need for orders of magnitude amplification and provide ubiquitous genotyping data, especially when amortizing multiple samples where sequencing reagent costs are reasonable. So, developing and improving NGS-based methods, such as multiple NGS-based methods called ApharSeq, is a good prospect for future research. As demonstrated in clinical samples, dozens of samples can be pooled early, reducing costs while providing variant information for each sample [78].

## 4. Protein-Based SARS-CoV-2 Variants Detection

Host infection with the SARS-CoV-2 virus produces structural and non-structural proteins, triggering an immune response that leads to the generation of viral protein-specific antibodies. Thus, viral proteins and/or specific antibodies against viral proteins can be used as detection targets to identify the presence of host infection with SARS-CoV-2 strains. For example, a protein-based target has been used widely in polymeric biosensor research to monitor anti-SARS-CoV-2 nucleocapsid protein monoclonal antibodies and diagnose COVID-19 [79]. At present, there are many detection technologies and kits for the S protein and N protein of SARS-CoV-2 [80,81,82], but due to the continuous mutation of SARS-CoV-2, the detection performance of these technologies needs to be tested, and methods need to be developed for these new mutations to identify variants and control the pandemic quickly.

### 4.1. Antigen-Based Detection

RAT plays an important role in detecting and controlling COVID-19 transmission, and while detection is less sensitive than RT-PCR, the advent of VOCs has also increased the need for RATs with high sensitivity and low detection limits [83,84,85]. Amazing COVID-19 antigen sealing tube test strip (Colloidal Gold) is based on the principle of the dual antibody sandwich method immunochromatographic assay, interpreting the results based on changes in the color of the bands provided in the test area (T) and control area (C). To verify the sensitivity and specificity of the Colloidal Gold for the diagnosis of the SARS-CoV-2 variants, 584 symptomatic and asymptomatic participants aged 0–90 years were enrolled, and the sensitivity to Delta/Kappa variants L452R and E484Q S gene mutations was 96.97% (95% CI, 84.24–99.92%), the sensitivity to mutations in the N501Y S gene of the Omicron variant was 90.80% (95% CI, 82.68–95.95%). This RAT has some sensitivity for the detection of the widely dispersed Omicron variant. The purpose of the test is to conduct self-testing at home and POC diagnosis and cannot yet be applied as a laboratory method to the detection of VOCs, but the results can strengthen community management of COVID-19 [86]. Moreover, the GeneFinder COVID-19 Ag Plus enables early diagnostic screening of SARS-CoV-2 to slow the spread of the virus. However, one study found that the GeneFinder COVID-19 Ag Plus rapid test was also able to identify Delta and Omicron BA.1 and BA.2 [87].

Therefore, in order to better understand the role of RATs in the detection of SARS-CoV-2 variants and achieve the purpose of early prevention of the COVID-19 outbreak, Table 2 shows the detection performance of RATs targeting N protein against SARS-CoV-2 variants due to the S protein being more prone to mutation.

Interestingly, despite the limited sensitivity of RAT, as an easy-to-use, accessible, and cost-effective assay, its sensitivity increased from 16.4% to 79.3% (Delta), between 28.2% and 89.5% (Omicron) over the course of consecutive assays [96,97]. Importantly, comparing the performance of immunochromatographic assay (ICA) and chemiluminescent enzyme immunoassay (CLEIA) in 111salivary samples found that RAT could be more effective against the Omicron variant than other SARS-CoV-2 variants, but this finding still needs to be confirmed by further study due to sample size [98].

### 4.2. Antibody-Based Detection

Enzyme-linked immunosorbent assays (ELISA) and LFA are common methods for detecting coronavirus proteins-specific antibodies. Variant detection has been studied using specific monoclonal antibodies that bind to specific antigens of SARS-CoV-2. For example, based on the change of N501Y in the SARS-CoV-2 variants, the recombinant monoclonal antibody design was carried out by using 2E8 obtained from patients diagnosed with COVID-19, and the N501 and Y501 in the spike protein RBD were distinguished by ELISA. That is, 2E8 bound to variants without N501Y mutations, such as Delta, not bound to Alpha, Beta, and Gamma, and then combined with recombinant CB6 mAb to distinguish Alpha and Gamma from Beta [99]. Secondly, mutations in key regions of primers or antibodies binding can affect the effectiveness of in vitro detection [100]; therefore, studies reported the development of specific monoclonal antibodies to detect the conservative epitope of SARS-CoV-2 N protein by lateral flow immunoassay (LFIA). Moreover, 75E12 as a capture probe and 54G6/54G10 combined as a detection probe could confirm SARS-CoV-2 infection in the context of the continuous evolution of SARS-CoV-2 mutations and avoid false-negative elevation due to mutations [101]. In addition, Alpha and Beta variants were successfully detected using anti-RBD antibodies (MD29 and MD65) as reporter antibodies, and anti-RBD antibody (BL6) and anti-NTD antibody (BL11) as capture antibodies [102]. It is difficult for immunoassay to detect antigenic variants of the emerging SARS-CoV-2 spike protein because spike protein mutations can alter viral antigenicity. Angiotensin-converting enzyme 2 (ACE2) is a receptor necessary for SARS-CoV-2 entry into host cells, and S protein mutations enhance the affinity for binding to the ACE2 receptor [103,104,105]. In using this mechanism, an ACE2 biosensor based on LFIA was designed, which was based on the binding target antigen principle of S1-mAb of red-labeled CNB and 180E11 antibody of blue-labeled CNB. Then SARS-CoV-2 wild type, S1 Alpha mutant type, S1 Beta mutant type, and neutralizing antibodies were detected according to the line intensity and color change after 20 min [106].

Taken together, since anti-spike protein mAb cloning technology has matured and generated a large amount of data on mAb-spike protein interaction, this type of method is well suited for POC detection for epidemiological monitoring and control. However, since the sensitivity of this assay needs to be improved, it is necessary to develop and select more specific monoclonal antibodies against SARS-CoV-2 variants for detection in future studies.

## 5. Combined Detection Technology

In order to make up for the lack of single detection technology, the application of some joint detection technologies has gradually developed. Firstly, considering the primer/probe binding site mismatches and false-negative results, there was research utilizing the principle of multiplex PCR-mass spectrometry minisequencing (mPCR-MS minisequencing). Multiplex PCR was used to amplify 9 mutations in 7 mutation sites of S gene HV69-70del, N501Y, K417N, P681H, D614G, E484K, L452R, P681R, and E484Q, and the extension mass probe was used to extend the SNP target site, MALDI-TOF MS was performed to detect the mass-to-charge ratio (m/z) of the mass probe extension (MPE) to determine the type of extended cardinality for identifying of the SARS-CoV-2 variants. Thus, this method could simultaneously identify six SARS-CoV-2 variants (Alpha, Beta, Epsilon, Iota, Gamma, and Delta), which had a specificity of 100% due to MPE detection, and the detection limit of all sites was between 100 and 400 copies, achieving multi-site simultaneous detection [107,108].

Secondly, the encodable multiplex microsphere phase amplification (MMPA) sensing platform, combined with primer-encoded microsphere technology and dual fluorescence decoding strategy, could detect SARS-CoV-2 RNA and VOCs with 4 h of turnaround time. The principle of the platform detection is as follows: first, the single nucleotide variants (SNVs) specific ARMS primer is coated to the surface of the microsphere so that the amplification refractory mutation system PCR (ARMS-PCR) reaction is fixed to the surface of a specific fluorescence-encoded microsphere by MMPA; second, the relationship between the microspheres and the SNV sites is determined by the specific fluorescent codes; and finally, the microsphere amplification enhancement is detected according to the fluorescence of the reported probe hybridized with the PCR products. It has a minimum detection limit of 28 copies/reaction of SARS-CoV-2 RNA and can recognize 10 key SNVs in the VOCs RBD, which solves the problems in multiplex PCR systems: non-specific amplification between primers and probes, and detection channels insufficiency [109].

In addition, the prevalence of COVID-19 has led to increasing demand for high-throughput, multiplexed, and sensitive detection methods. The Microfluidic Combination Array Reaction (mCARMEN), which combined CRISPR/Cas12/Cas13-based diagnostics and microfluidics technology, could rapidly detect Alpha, Beta, Gamma, Delta, Epsilon, and Omicron while quantifying 5–6 orders of magnitude of the viral copy number of SARS-CoV-2 in the sample, distinguishing these mutations from VOCs that require future attention. In order to meet the needs of clinical testing, the mCARMEN workflow has been further streamlined. To this end, a series of steps were implemented to reduce the processing time from >8 h to <5 h, such as automated RNA extraction, RNA to DNA amplification with 1 primer pool, and reduced duration of detection results. As the only diagnostic method that combines monitoring functions into a single technology platform, mCARMEN is highly scalable and more comprehensive than nucleic acid-based diagnosis [110].

In short, the combined detection technology makes full use of the advantages of each detection method to maximize the sensitivity and specificity of the relatively optimal, more cost-effective, simpler workflow, and it is more suitable for clinical applications.

## 6. Conclusions and Perspectives

Most tests for the SARS-CoV-2 variants are based on nucleic acid or protein assays. PCR-based detection can only quickly identify variants with known mutation characteristics, not emerging variants, so WGS is still the gold standard for determining new SARS-CoV-2 variants. Protein detection, on the other hand, is an alternative and necessary complementary method to nucleic acid detection, which can supplement epidemiological information that is not available by other methods. Table 3 summarizes the advantages and disadvantages of various SARS-CoV-2 VOCs testing methods.

In conclusion, this review summarized and analyzed the latest advances in the detection methods of VOCs. It is helpful for further controlling the epidemic of COVID-19, quickly applying the best detection methods, and then taking corresponding health surveillance, isolation, and treatment measures for SARS-CoV-2 variants and their possible new variants in the future. In addition, future research of SARS-CoV-2 variants should focus more on developing and improving portable, self-testing, mutation site specificity, accurate, harmless, rapid, and easy-to-observe, and interpretable results methods of detection. It can reduce the working hours and labor intensity of healthcare workers and also meet the needs of family self-testing.

## Figures and Tables

**Table 1:** Summary of Mutation-specific PCR Methods for Detecting SARS-CoV-2 VOCs.

SARS-CoV-2 Variants	Mutation-Specific Targets	References
Alpha	ΔH69/ΔV70 + N501Y	[14]
Alpha; Beta; Gamma	N501Y + delHV69/70; N501Y + K417N; N501Y + V1176F	[15]
Alpha; Beta	S.1.1.7; B.241–243	[16]
Alpha; Beta; Gamma	L452R + D614G; L5F + L18F + Δ69–70 + D80 + Δ144 + E484K + N501Y + D614G + P681H; D80 + Δ241-3 + K417N + N501Y + D614G; L18F + T20N + P26S + D138Y + K417T + E484K + N501Y + D614G + V1176F	[17]
Gamma; Delta	four-nucleotide insertion of ORF8; SΔ157–158	[18]
Alpha; Beta; Gamma	S69–70del + SN501Y + ND3L; SN501Y	[19]
Alpha; Beta; Gamma; Delta	N501Y + HV69/70del + E484; N501Y + E484K + K417N; N501Y + E484K + K417T + V1176F; N501 + E484 + (L452R or P681R or T478K)	[20]
Alpha; Beta; Gamma	HV69/70 + N501Y; N501Y + E484K;	[21]
Alpha; Beta; Gamma; Delta	P314L + RG203_204KR + N501Y; P314L + N501Y + E484K + K417N + A701V; P314L + RG203_204KR + N501Y + E484K + V1176F; P314L + L452R	[22]
Alpha; Delta	C1709A; C56G	[23]
Omicron BA.1	SGTF +ΔH69/ΔV70 + K417N	[24]
Omicron BA.1; Omicron BA.2	Q493R + Q498R + G496S; Q493R + Q498R	[25]
Alpha; Beta; Gamma; Delta; Omicron	N501Y +ΔH69/ΔV70; N501Y + E484K + K417N; N501Y + E484K + K417T; L452R + P681R + T478K; N501Y +ΔH69/ΔV70 + T478K	[26]
Alpha; Beta + Gamma; Delta; Omicron	N501Y + ΔH69/V70 + E gene; 242–244 WT + K417N + E484K + K417T; L452R + P681 WT + E484Q; N501Y + ΔH69/V70 + E gene + 242-244 WT + K417N	[27]

**Table 2:** Detection Performance of Various RAT Kits for SARS-CoV-2 Variants.

Samples	Test Kits	SARS-CoV-2 Variants	Detection Performance	References
Infectious Vero E6 cell culture supernatants	SARS-CoV-2 Rapid Antigen Test (Roche)	Alpha	LOD in RNA copies/mL: 8.9 × 10^5^ (DMEM) and 1.9 × 10^6^ (saliva)	[88]
		Beta	LOD in RNA copies/mL: 8.5 × 10^5^ (DMEM) and 1.1 × 10^6^ (saliva)	
	CLINITEST Rapid COVID-19 Antigen Test (Siemens)	Alpha	LOD in RNA copies/mL: 8.9 × 10^5^ (DMEM) and 1.9 × 10^6^ (saliva)	
		Beta	LOD in RNA copies/mL: 8.5 × 10^5^ (DMEM) and 1.1 × 10^6^ (saliva)	
	Panbio COVID-19 Ag RAPID TEST DEVICE (Abbott)	Alpha	LOD in RNA copies/mL: 8.9 × 10^5^ (DMEM) and 1.9 × 10^6^ (saliva)	
		Beta	LOD in RNA copies/mL: 8.5 × 10^5^ (DMEM) and 1.1 × 10^6^ (saliva)	
	NADAL COVID-19 Ag rapid test (nal von minden)	Alpha	LOD in RNA copies/mL: 8.9 × 10^5^ (DMEM) and 1.9 × 10^6^ (saliva)	
		Beta	LOD in RNA copies/mL: 8.5 × 10^5^ (DMEM) and 6.4 × 10^6^ (saliva)	
	BIOCREDIT COVID-19 Ag rapid test kit (RapiGEN)	Alpha	LOD in RNA copies/mL: 3.4 × 10^7^ (DMEM) and 6.6 × 10^7^ (saliva)	
		Beta	LOD in RNA copies/mL: 2.6 × 10^7^ (DMEM) and 3.9 × 10^7^ (saliva)	
A total of 410 respiratory samples	Lumipulse G SARS-CoV-2 Ag (CLEIA)	Alpha	Viral loads: 1 × 10^5^ Geq/mL	[89]
		Beta	Viral loads: 1 × 10^5^ Geq/mL	
	Elecsys SARS-CoV-2 Ag (ECLIA)	Alpha	Viral loads: 5 × 10^5^ Geq/mL	
		Beta	Viral loads: 2 × 10^6^ Geq/mL	
	LIAISON SARS-CoV-2 Ag (CLIA)	Alpha	Viral loads: 8 × 10^6^ Geq/mL	
		Beta	Viral loads: 8 × 10^6^ Geq/mL	
	SARS-CoV-2 Ag ELISA (ELISA)	Alpha	Viral loads: 8 × 10^6^ Geq/mL	
		Beta	Viral loads: 8 × 10^6^ Geq/mL	
Cell culture medium and pooled saliva	SARS-CoV-2 Rapid Antigen Test	Alpha	LOD in RNA copies/mL: 1 × 10^6^ (DMEM) and 1 × 10^6^ (saliva)	[90]
		Beta	LOD in RNA copies/mL: 2 × 10^6^ (DMEM) and 2 × 10^6^ (saliva)	
		Gamma	LOD in RNA copies/mL: 1 × 10^6^ (DMEM) and 1 × 10^6^ (saliva)	
		Delta	LOD in RNA copies/mL: 2 × 10^6^ (DMEM) and 2 × 10^6^ (saliva)	
	CLINITEST Rapid COVID-19 Antigen Self-Test	Alpha	LOD in RNA copies/mL: 1 × 10^6^ (DMEM) and 2 × 10^6^ (saliva)	
		Beta	LOD in RNA copies/mL: 1 × 10^5^ (DMEM) and 1 × 10^5^ (saliva)	
		Gamma	LOD in RNA copies/mL: 1 × 10^6^ (DMEM) and 1 × 10^6^ (saliva)	
		Delta	LOD in RNA copies/mL: 1 × 10^6^ (DMEM) and 1 × 10^6^ (saliva)	
	Rapid SARS-CoV-2 Antigen Test Card	Alpha	LOD in RNA copies/mL: 1 × 10^5^ (DMEM) and 1 × 10^6^ (saliva)	
		Beta	LOD in RNA copies/mL: 1 × 10^5^ (DMEM) and 2 × 10^6^ (saliva)	
		Gamma	LOD in RNA copies/mL: 2 × 10^6^ (DMEM) and 2 × 10^6^ (saliva)	
		Delta	LOD in RNA copies/mL: 1 × 10^6^ (DMEM) and 1 × 10^6^ (saliva)	
	Panbio COVID-19 Ag RAPID TEST DEVICE	Alpha	LOD in RNA copies/mL: 2 × 10^6^ (DMEM) and 2 × 10^6^ (saliva)	
		Beta	LOD in RNA copies/mL: 2 × 10^6^ (DMEM) and 1 × 10^5^ (saliva)	
		Gamma	LOD in RNA copies/mL: 1×10^6^ (DMEM) and 2×10^6^ (saliva)	
		Delta	LOD in RNA copies/mL: 2 × 10^6^ (DMEM) and 2 × 10^6^ (saliva)	
55 nasopharyngeal swab samples	SD BIOSENSOR	Beta, Gamma	Sensitivity (a positive group diagnosed with the SARS-CoV-2 variants by RT-qPCR): 42.8% with RT-qPCR amplification range 20 ≤ Cq < 25	[91]
319 nasopharyngeal specimens	the KestrelTM COVID-19 Ag Rapid Test	Alpha	LOD: 0.156 ng/mL	[92]
		Beta	LOD: 0.156 ng/mL	
		Gamma	LOD: 0.156 ng/mL	
		Delta	LOD: 0.156 ng/mL	
		Epsilon	LOD: 0.156 ng/mL	
		Kappa	LOD: 0.156 ng/mL	
		Omicron	LOD: 0.39 ng/mL	
VeroE6 cells and four six-week-old Syrian golden hamsters	OraSure InteliSwab™ Rapid Antigen Test	Alpha	LOD: 0.313 ng/mL, genome Copies/mL: 6.06 × 10^5^	[93]
		Beta	LOD: 0.469 ng/mL, genome Copies/mL: 3.77 × 10^5^	
		Gamma	LOD: 0.313 ng/mL, genome Copies/mL: 4.30 × 10^5^	
		Delta	LOD: 0.469 ng/mL, genome Copies/mL: 9.13 × 10^5^	
		Omicron	LOD: 0.469 ng/mL, genome Copies/mL: 4.51 × 10^5^	
SARS-CoV-2 heat-inactivated positive samples	The Abbott BinaxNOW SARS-CoV-2 rapid antigen test	Alpha	Lowest viral load (highest RT-qPCR Ct value): 28.9	[94]
		Beta	Lowest viral load (highest RT-qPCR Ct value): 25.92	
		Gamma	Lowest viral load (highest RT-qPCR Ct value): 26.14	
		Delta	Lowest viral load (highest RT-qPCR Ct value): 26.7	
		Eta	Lowest viral load (highest RT-qPCR Ct value): 26.28	
		Lambda	Lowest viral load (highest RT-qPCR Ct value): 23.81	
		Mu	Lowest viral load (highest RT-qPCR Ct value): 24.25	
		Omicron	Lowest viral load (highest RT-qPCR Ct value): 24.6	
10 authentic SARS-CoV-2 variants, and 148 symptomatic clinical samples	CoV-SCAN	Alpha	LOD: 6.25 TCID50/swab	[95]
		Beta	LOD: 12.5 TCID50/swab	
		Gamma	LOD: 12.5 TCID50/swab	
		Delta	LOD: 6.25 TCID50/swab	
		Omicron	LOD: 3.2 TCID50/swab; Sensitivity (a positive group diagnosed with the SARS-CoV-2 variants by RT-qPCR): 93.8%	

**Table 3:** Comparison of Different SARS-CoV-2 VOCs Detection Methods.

Methods	Advantages	Disadvantages
Mutation-specific SARS-CoV-2 PCR/Multiplex PCR	Short turnaround times; 98.6% sensitivity; reduce the number of reagents required for sample analysis and hands-on time; simultaneous detection of multiple mutation sites	The genetic information provided is limited; the mutant nature of the SARS-CoV-2 variant needs to be known
LAMP	Sensitivity of 90% or more; simple operation; fast amplification speed; high efficiency; no need for complex thermal cycler; can be used at the grass-roots level, laboratories with poor experimental conditions, and POC	False-positive; cross-contamination; poor stability
CRISPR-Cas-based detection technology	100.0% specificity and accuracy; rapid screening; low cost; suitable for POC diagnosis	Only for point mutations; relatively low sensitivity to samples with low viral load 53.97%
RT-ddPCR	Absolutely quantitative; sensitive and accurate	Expensive
RAT	Short turnaround times; easy to use; cost-effective; no complex instrumentation and/or expertise required for results interpretation; suitable for home inspection analysis	False-negative; sensitivity is not high compared to nucleic acid detection-based; particularly sensitive to sample quality
ELISA	High negative predictive value; high throughput suitable for POC detection	Low sensitivity due to large differences in affinity between variants; no clinically implemented testing
LFA	Specificity; long-term stability; suitable for home testing and self-testing	Low sensitivity due to the identification of only specific regions of the antigen
Viral genome sequencing	High-throughput sequencing; super sensitive; provide a detailed map of new mutations; accurately identify mutation types	Resource-intensive; long turnaround times of hours to weeks; expensive; demanding in technic and equipment

## Data Availability

Not applicable.
